# Endothelial function is unaffected by changing between carvedilol and metoprolol in patients with heart failure-a randomized study

**DOI:** 10.1186/1475-2840-10-91

**Published:** 2011-10-15

**Authors:** Britt Falskov, Thomas Steffen Hermann, Jakob Raunsø, Buris Christiansen, Christian Rask-Madsen, Atheline Major-Pedersen, Lars Køber, Christian Torp-Pedersen, Helena Dominguez

**Affiliations:** 1 Department of Cardiology, Gentofte Hospital, Hellerup, Denmark; 2 Department of Cardiology, Herlev Hospital, Herlev, Denmark; 3 Joslin Diabetes center, Boston(MA), USA; 4 Department of Cardiology, Rigshospitalet Heart Center, Copenhagen

**Keywords:** Heart failure, Endothelial function, Beta blocker

## Abstract

**Background:**

Carvedilol has been shown to be superior to metoprolol tartrate to improve clinical outcomes in patients with heart failure (HF), yet the mechanisms responsible for these differences remain unclear. We examined if there were differences in endothelial function, insulin stimulated endothelial function, 24 hour ambulatory blood pressure and heart rate during treatment with carvedilol, metoprolol tartrate and metoprolol succinate in patients with HF.

**Methods:**

Twenty-seven patients with mild HF, all initially treated with carvedilol, were randomized to a two-month treatment with carvedilol, metoprolol tartrate or metoprolol succinate. Venous occlusion plethysmography, 24-hour blood pressure and heart rate measurements were done before and after a two-month treatment period.

**Results:**

Endothelium-dependent vasodilatation was not affected by changing from carvedilol to either metoprolol tartrate or metoprolol succinate. The relative forearm blood flow at the highest dose of serotonin was 2.42 ± 0.33 in the carvedilol group at baseline and 2.14 ± 0.24 after two months continuation of carvedilol (P = 0.34); 2.57 ± 0.33 before metoprolol tartrate treatment and 2.42 ± 0.55 after treatment (p = 0.74) and in the metoprolol succinate group 1.82 ± 0.29 and 2.10 ± 0.37 before and after treatment, respectively (p = 0.27). Diurnal blood pressures as well as heart rate were also unchanged by changing from carvedilol to metoprolol tartrate or metoprolol succinate.

**Conclusion:**

Endothelial function remained unchanged when switching the beta blocker treatment from carvedilol to either metoprolol tartrate or metoprolol succinate in this study, where blood pressure and heart rate also remained unchanged in patients with mild HF.

**Trial registration:**

Current Controlled Trials NCT00497003

## Introduction

Beta blocker treatment is a well-established therapy for heart failure (HF), but the drugs tested have different profiles of possible clinical consequence. In the Carvedilol Or Metoprolol European Trial (COMET) treatment with carvedilol was found superior in patients with chronic heart failure when compared to metoprolol tartrate [1].

Patients with heart failure are characterized by having an impaired endothelial function regardless of the etiology of heart failure [2]. An impaired endothelial function in patients with heart failure is associated with a poor prognosis [3,4] and the severity of endothelial function in HF is proportional to the New York Heart Association heart failure classification (NYHA) in HF [3]. We therefore designed this study to investigate whether metoprolol tartrate, metoprolol succinate and carvedilol respectively affect vascular endothelial function and vascular insulin resistance differently in patients with chronic heart failure.

Patients with HF are at increased risk of developing diabetes and frequently demonstrate insulin resistance [5,6]. In the Carvedilol Or Metoprolol European Trial (Comet) study it was observed that metoprolol deteriorates metabolic glucose control whereas carvedilol does not [7] and, accordingly, there was a larger number of new-onset diabetes in patients treated with metoprolol [8]. We have observed that vascular insulin sensitivity was deteriorated after treatment with metoprolol in patients with type 2 diabetes, whereas no change was found after treatment with carvedilol [9]. We hypothesized that changes in endothelial insulin sensitivity might be involved in the processes by which survival is different during treatment with carvedilol and metoprolol.

The beta-1 adrenergic receptor blocking effect of metoprolol tartrate has been doubted to be as effective as the beta-1 adrenergic blocking effect of both carvedilol and metoprolol succinate [10]. We therefore performed a 24-hour ambulatory blood pressure measurement as well as heart rate measurements in patients receiving either carvedilol, metoprolol tartrate or metoprolol succinate to evaluate potential differences in the adrenergic effects among three treatment groups. We aimed to find potential differences among beta blockers, when used in recommended doses, to obtain what is thought to be equivalent treatment doses.

## Method and materials

### Population

Thirty patients with mild heart failure (HF) were included in the study. Inclusion criteria were heart failure with a Left Ventricular Ejection Fraction (LVEF) of 35% or below, documented by echocardiography at the time of entering the study and stable NYHA class I-II. Decompensated heart failure, beta blocker intolerance, uncontrolled hypertension, hypotension and bradycardia were all exclusion criteria for entering the study. Patients were secondarily excluded from the study if they became clinically unstable or had to change medical treatment during the study period. Ten volunteers with no documented cardiovascular disease, no diabetes and no medication use were enrolled as control group for comparison on baseline measurements.

Patient recruitment was done by advertisement in newspapers and from an out-patients clinic. Informed consent was given before entering the study. The study was approved by the ethics committee of the city of Copenhagen (ref KF 02-071/03), as well as the Danish Medicines Agency (ref 2612-2423) and registered at clinical trials.gov (ref NCT00497003).

### Design

Before randomization all patients were treated with carvedilol for at period of at least two months to ensure equal use of beta blockers at baseline. Patients receiving beta blocker treatment at the time they were included in the study, had their beta blocker treatment changed to carvedilol. If the patients were beta blocker naïve at the time of inclusion, they started treatment with carvedilol and titrated to the highest tolerable dose. The patients were then randomized to receive treatment with carvedilol, metoprolol tartrate or metoprolol succinate for a period of two months. The group of patients who continued on carvedilol treatment served as time-control. Patients were otherwise kept on their usual medication throughout the whole study period.

Ten patients were randomized to receive carvedilol with a target dose of 50 mg a day; ten patients were randomized to receive metoprolol succinate with a target dose of 200 mg a day and ten patients were randomized to receive metoprolol tartrate with a target dose of 200 mg a day.

The study was designed as an open parallel group study. Before and after the two months randomization period an examination of endothelial function as well as insulin stimulated endothelial function was performed by venous occlusion plethysmography as described in the following section. Forearm glucose uptake was measured during intra-arterial insulin infusion both before and after the treatment period. 24 hour blood pressure and heart rate measurements were also done before and after the two months randomization period.

### Venous occlusion plethysmography

Endothelial function was measured by using the method venous occlusion plethysmography as described before [11]. Venous occlusion plethysmography is an invasive technique in which vasoactive agents are infused directly into the artery. The technique allows us to test a vasoactive response to different agents without systemic changes. Additionally we tested the vasoactive properties of insulin as well as direct insulin stimulated glucose-uptake in the forearm. Venous occlusion plethysmography has been found to be a valid method of measuring endothelial function [12].

Examinations were done after an overnight fast, in a quiet room with the temperature kept constant during the day. The patients were all abstinent from smoking for at least 8 hours and did not take their usual medication in the morning before examination. Examinations were done with the patients lying supine with the forearm at a horizontal level with the right atrium.

Endothelium-dependent vasodilatation was assessed stepwise after an intra-arterial infusion of increasing doses of serotonin (7, 21, 70 ng/min) [Serotonin (Clinalfa, Läufelfingen, Switzerland)] for 4 minutes at each dose level to achieve a dose-response profile. Serotonin is an agonist of endothelial nitric oxide (NO) production. Endothelium-independent vasodilatation was assessed by infusion of increasing doses of nitroprusside [Nitropress (Abbott Laboratories, North Chicago, IL)] (0.5, 1 and 1.5 μg/min). Nitroprusside is an external NO donor in vascular smooth muscle cells and provide vascular smooth muscle relaxation and dilatation. To assess insulin-stimulated endothelial vasodilatation, insulin [Actrapid (Novo Nordisk Scandinavia, Malmö Sweden) in a 1% human albumin solution (vehicle)] was co-infused with serotonin. Insulin was infused intra-arterially for 60 minutes prior to the vaso-reactivity studies with serotonin, at a rate of 0.05 mU/kg body weight/min. Studies with co-infusions of N^G^-monomethyl-L-arginine [L-NMMA (Clinalfa, Läufelfingen, Switzerland)], serotonin and insulin were done to determine the NO-dependent fraction of the insulin-stimulated endothelium-dependent vasodilatation. L-NMMA is a non-specific NO synthase inhibitor. Flow measurements are presented as the relative blood flow, given as a proportion between the actual blood flow (ml/min) in the infused arm and the actual blood flow in the non-infused arm. All evaluation of blood flow was performed blinded to treatment allocation.

### Forearm Glucose uptake

Blood samples were drawn simultaneously from a catheter placed intravenously in the infused arm, a catheter placed in the brachial artery of the infused arm, and a catheter placed intravenously in the non-infused arm. The latter served as a control for systemic changes in concentrations of insulin and glucose during intra-arterial infusion of insulin. Forearm glucose uptake was calculated as the arterio-venous difference in glucose concentration in relation to forearm blood flow in samples from the artery and vein of the infused arm, respectively, and done on both examination days-before and after the two-month treatment period with either of the three beta blockers. Plasma glucose concentrations were determined by the glucose oxidase method [Vitros Chemistry; Johnson & Johnson, Rochester New York] and serum insulin concentrations by a chemiluminescent immunometric assay [Immulite 2500; DPC, USA].

Measurements of systemic metabolic changes of insulin resistance were calculated with the use of a computerized Homeostatic model assessment calculator [(HOMA2) Diabetes Trial Units, The Oxford Centre for Diabetes, Endocrinology and Metabolism]. The used formula for the calculations: HOMA Insulin Resistance = (Fasting Plasma Insulin (mU/L) × Fasting Plasma Glucose (mmol/L))/22.5.

### 24 hour ambulatory blood pressure and measurements of heart rate

Changes in blood pressure and heart rate were examined in a sub-group of patients in all three treatment groups. Patients had a 24-hour ambulatory blood pressure and heart rate examination done on a separate day before and after the randomized treatment period. An ambulatory blood pressure monitor was used [Model TM-2430; A&D Instruments ltd., Oxford, UK] and computer software was used for retrieving data [EZ Doctor Software for TM-2430; Kivex A/S, Hørsholm, Denmark].

### Statistics

Results are expressed as means ± standard error of mean (SEM), unless otherwise specified.

Comparisons of baseline differences between the groups were performed using unpaired Students t-test. Paired comparisons after the treatment or observation period were analyzed with students paired t-test. Differences between groups for single parameters after the treatment with either of the three beta blockers were compared with 2-way analysis of variance (ANOVA). Changes in forearm blood flow as well as changes in forearm glucose uptake were subject to analysis of variance for repeated measurements using the proc mixed procedure in the Statistical Analyses Software, version 8.0 (SAS Institute, Cary, NC, USA). Forearm glucose uptake measurements were log transformed to satisfy assumptions of normal distribution and homogeneity of variance of residuals. Subjects entered the model as random effect. The dose of vasodilator and the interaction between insulin and serotonin entered the model as fixed values.

Multivariable association between flow, treatment and other cofactors was examined in mixed variance covariance models using proc mixed from SAS. Individual and interaction between study and individual were random variables whereas cofactors and interaction between study day and treatment were entered as fixed variables.

From previous studies in our group [11] it was found that a sample size of 10 patients in each group, a difference of 20% in forearm blood flow and forearm glucose uptake can be found with a statistical significance of 5%.

## Results

Both men and women were included in the study and they were at the age 46 to 80 years old at the time the study took place. Three patients were excluded from the study after randomization, one in each treatment group. Two patients were excluded due to difficulties in cannulation at the second examination day and one patient was excluded because of an episode of unstable angina during the treatment period. Therefore nine patients in each treatment group completed the study.

The carvedilol doses achieved at baseline were similar in the three groups: 37.2 ± 5.3 mg a day in the group that was randomized to continue on carvedilol, 36.8 ± 5.6 mg a day in the group that was switched to metoprolol succinate treatment and 34.0 ± 6.5 mg a day in the group that was switched to metoprolol tartrate. After randomization and through the two-month treatment period, the doses of the respective treatments were as follows: 37.2 ± 5.3 mg a day of carvedilol, 147.2 ± 22.2 mg of metoprolol succinate and 130.6 ± 24.9 mg a day of metoprolol tartrate.

Baseline data and changes after the two-month treatment period are shown in table 1 for all three treatment groups as well as baseline data for the healthy control group. Nine, seven and four patients in the carvedilol, metoprolol succinate and metoprolol tartrate group respectively, received either an angiotensin-converting enzyme (ACE) inhibitor or an angiotensin II receptor antagonist (AT II antagonist). The use of statins as well as aspirin was equal in all three treatment groups. After initial screening, patients who were using other beta blockers than carvedilol were changed to carvedilol and those who where not using any, were initiated to treatment with carvedilol and titrated to the highest tolerable dose.

**Table 1:** Baseline characteristics of the patients randomized to carvedilol, metoprolol succinate and metoprolol tartrate before and after the treatment period, and baseline characteristics of the healthy control group

	Carvedilol (N = 9)		Metoprolol Succinate (N = 9)		Metoprolol Tartrate (N = 9)		Healthy controls (N = 10)
		
	Before treatment	After treatment	Before treatment	After trreatment	Before treatment	After treatment	
**Age**	67.1 ± 3.31		63.6 ± 2.02		60.3 ± 3.26		47.6 ± 1.89

**Sex (Female/Male)**	2/7		2/7		1/8		5/5

**Smokers (Y/N)**	4/5		1/8		2/7		0

**Diabetes (Y/N)**	3/6		3/6		0/9		0

**NYHA I (N°)**	5		4		5		0

**NYHA II (N°)**	4		5		4		0

**ACE I/ATII ant (Y/N)**	9/0		7/2		5/4		0

**Aspirin (Y/N)**	6/3		6/3		7/2		0

**Statins (Y/N)**	7/2		8/1		7/2		0

**CRP**	< 10	< 10	< 10	< 10	< 10	< 10	< 10

**Hgb (mmol/l)**	8.1 ± 0.30	7.97 ± 0.34	8.48 ± 0.23	8.37 ± 0.33	8.54 ± 0.19	8.47 ± 0.16	8.00 ± 0.21

**Total Cholesterol (mmol/l)**	3.88 ± 0.23	3.89 ± 0.30	4.21 ± 0.27	3.96 ± 0.22	3.88 ± 0.18	3.81 ± 0.19	4.5 ± 0.29

**Triglyceride (mmol/l)**	1.57 ± 0.40	1.35 ± 0.20	1.66 ± 0.41	1.74 ± 0.31	1.57 ± 0.38	1.57 ± 0.36	0.9 ± 0.17

**HDL (mmol/l)**	1.47 ± 0.20	1.24 ± 0.20^3^	1.44 ± 0.16	1.15 ± 0.11^3^	1.34 ± 0.12	1.27 ± 0.13	1.4 ± 0.14

**LDL (mmol/l)**	1.88 ± 0.17	2.03 ± 0.24	2.24 ± 0.31	2.03 ± 0.24	1.80 ± 0.24	1.88 ± 0.27	2.7 ± 0.26

**Hgb A1c (%)**	6.12 ± 0.20	5.91 ± 0.23^3^	6.78 ± 0.49	6.73 ± 0.51	5.64 ± 0.10¹^2^	5.5 ± 0.25^2^	5.24 ± 0.10

**Plasma-Glucose**	6.30 ± 0.29	6.24 ± 0.69	5.73 ± 0.42	5.82 ± 0.49	5.19 ± 0.26¹	5.14 ± 0.31	

**Serum-Insulin**	24.80 ± 11.72	27.50 ± 12.96	9.72 ± 2.52	17.67 ± 4.71	8.52 ± 2.93	8.32 ± 3.48	

**HOMA-IR**	1.86 ± 0.67	1.77 ± 0.37	1.33 ± 0.34	1.87 ± 0.62	1.11 ± 0.40	1.33 ± 0.56	

**Body weight (kg)**	77.58 ± 6.60	77.47 ± 6.74	90.21 ± 6.17	90.19 ± 6.40	86.82 ± 3.53	87.59 ± 3.99	75.62 ± 4.24

**BMI (kg/m^2^)**	26.37 ± 1.23	26.32 ± 1.27	29.41 ± 1.63	29.38 ± 1.65	28.1 ± 0.84	28.31 ± 0.98	24.4 ± 0.93

¹p < 0.05 for comparison between metoprolol tartrate and carvedilol

^2^p < 0.05 for comparison between metoprolol tartrate and metoprolol succinate

^3^p < 0.05 change from before and after randomization treatment

Before randomization, patients in the metoprolol tartrate group had a lower fasting glucose and HgbA1c than the other treatment groups (p < 0.05) (table 1). This could be explained by the fact that there were three patients with type 2 diabetes included in the carvedilol as well as the metoprolol succinate group and none in the metoprolol tartrate group. In the carvedilol group glycosylated hemoglobin (HgbA1c) was also lower after the two months treatment period (p < 0.01).

Patients in the metoprolol succinate as well as the time control group-the group kept on carvedilol treatment-tended to have a lower high-density lipoprotein (HDL) level after the two months treatment. In the carvedilol group the HDL level was 1.47 ± 0.20 mmol/l before randomization and 1.24 ± 0.20 mmol/l two months after randomisation (p < 0.05). In the metoprolol succinate group, HDL level changed from 1.44 ± 0.16 mmol/l to 1.15 ± 0.11 before and after randomization, respectively (p < 0.05). HDL level in the metoprolol tartrate group was unchanged through the study period.

### Vaso-reactivity studies

**Figure 1:**
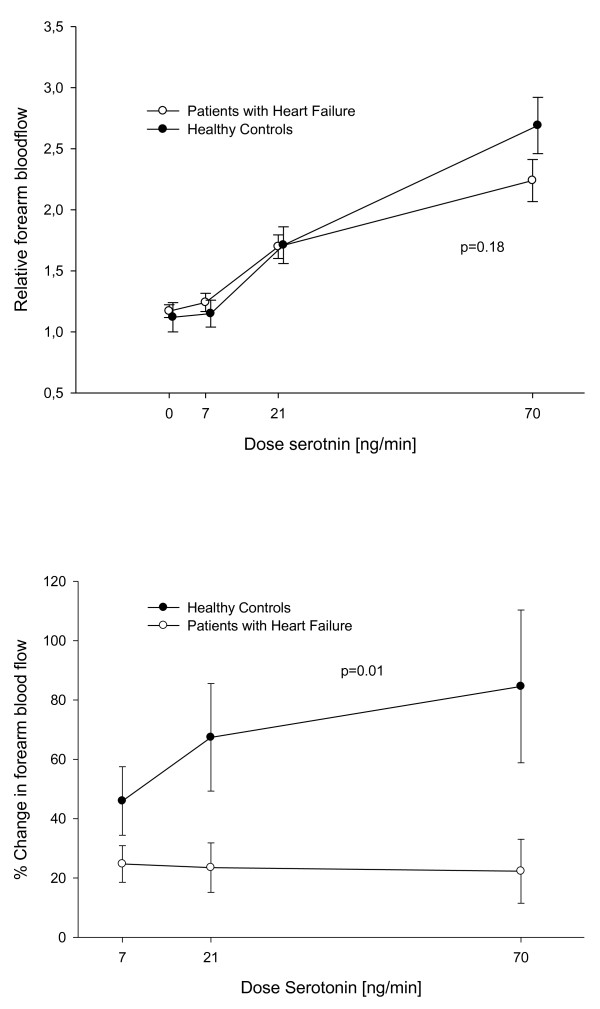
**Baseline flow**. Comparison of the forearm blood flow response and the percentage change in endothelial function after infusion of Insulin between patients with heart failure and healthy controls respectively.

The group of patients with HF had a slightly but not significantly lower response to serotonin compared to the group of healthy controls (Figure 1a), while insulin-stimulated endothelial function was markedly reduced in this group (Figure 1b).

**Figure 2:**
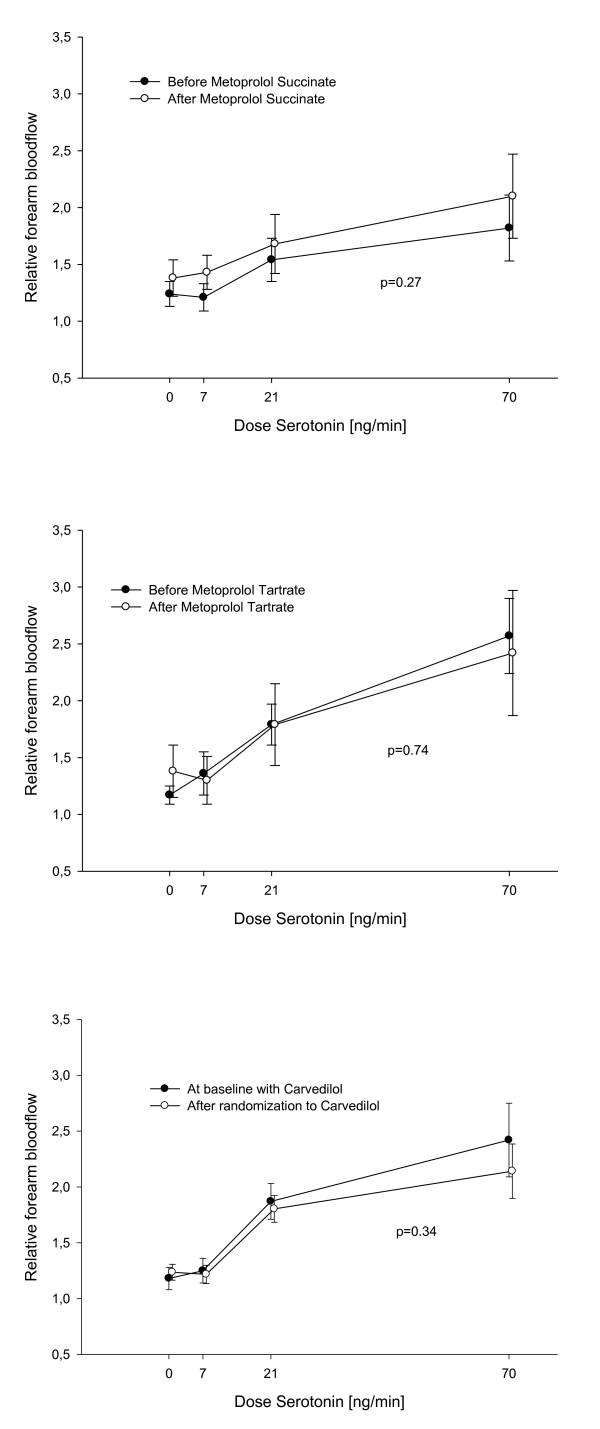
**Serotonin response**. Comparison of the forearm blood flow response before and after the treatment period with metoprolol succinate, metoprolol tartrate and carvedilol respectively.

Endothelium-dependent vasodilatation assessed with infusion of increasing doses of serotonin was equal in the three groups before randomization and did not change after switching beta blocker treatment from carvedilol to metoprolol succinate or metoprolol tartrate: In the group randomized to metoprolol succinate the relative forearm blood flow was 1.24 ± 0.11; 1.21 ± 0.12; 1.54 ± 0.19; and 1.82 ± 0.29 at each dose level of serotonin before randomization treatment and 1.38 ± 0.16; 1.43 ± 0.15; 1.68 ± 0.26; and 2.10 ± 0.37 after two months treatment with metoprolol succinate (p = 0.27) (Figure 2a). In the group randomized to metoprolol tartrate the relative blood flow was 1.17 ± 0.08; 1.36 ± 0.19; 1.79 ± 0.18 and 2.57 ± 0.33 before randomization and 1.38 ± 0.23; 1.30 ± 0.21; 1.79 ± 0.34 and 2.42 ± 0.55 after the treatment period (p = 0.74) (Figure 2b). The third group was kept on carvedilol treatment after randomization and blood flow did not change during the treatment period: The blood flow was 1.18 ± 0.10; 1.25 ± 0.11; 1.87 ± 0.16 and 2.42 ± 0.33 before randomization and 1.24 ± 0.07; 1.22 ± 0.08; 1.80 ± 0.12 and 2.14 ± 0.24 after randomization treatment period (p = 0.34) (Figure 2c).

**Figure 3:**
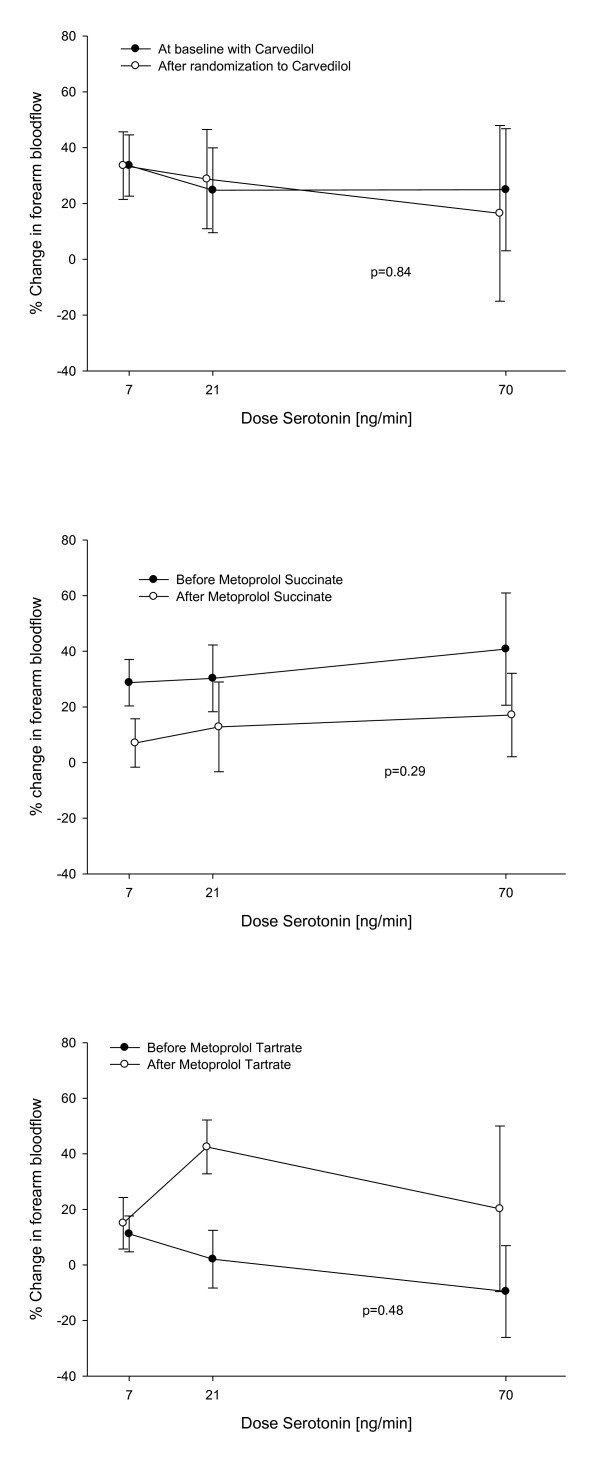
**Insulin-stimulated endothelial function**. Percentage change in forearm bloodflow after co-infusion of Insulin, before and after the treatment period with carvedilol, metoprolol succinate and metoprolol tartrate respectively.

Switching beta blocker treatment did not affect insulin-stimulated endothelial function significantly: The percentage change in forearm blood flow in patients randomized to stay on carvedilol treatment for each serotonin dose was 33.58% ± 10.97; 24.74% ± 15.21 and 24.92% ± 21.86 before randomization and 33.56% ± 12.10; 28.72% ± 17.77 and 16.45% ± 31.46 after the treatment period (p = 0.84) (Figure 3a). In the metoprolol succinate group the percentage change in flow was 28.71% ± 8.36; 10.27% ± 12.00 and 40.78% ± 20.19 before treatment with metoprolol succinate and 7.03% ± 8.69; 12.82% ± 16.13 and 17.09% ± 15.01 after two months treatment (p = 0.29) (Figure 3b). In the metoprolol tartrate group the percentage change in flow was 11.19% ± 6.45; 2.08% ± 10.38 and -9.55% ± 16.51 before randomization and 15.03% ± 9.26; 42.48% ± 9.69 and 20.22% ± 29.79 after treatment (p = 0.48) (Figure 3c).

Studies of endothelium-independent vasodilatation with sodium nitroprusside were also not affected by the change in beta blocker treatment (data not shown). L-NMMA co-infusion with both insulin and serotonin abolished the stimulated vasodilatation and was unchanged in the three treatment groups (data not shown).

### Other analysis

Multivariable analysis of the relation between treatment (carvedilol, metoprolol tartrate and metoprolol succinate) and serotonin induced flow also revealed no relation when diabetes, ACE inhibitor or AT antagonist treatment, treatment with statins, smoking, NYHA class, left ventricular ejection fraction (LVEF) and body mass index (BMI) were also entered in the analysis (p = 0.36). Also insulin induced flow changes were not changed by treatment when tested with multivariable analysis (p = 0.32).

### Forearm glucose uptake

**Figure 4:**
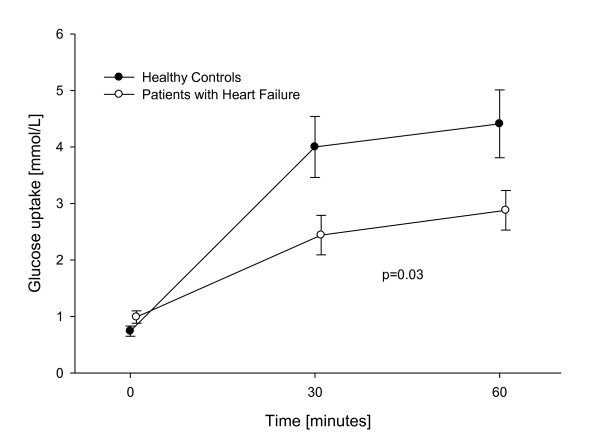
**Baseline forearm glucose uptake**. Forearm glucose uptake during insulin infusion. Patients with heart failure compared to healthy controls.

**Figure 5:**
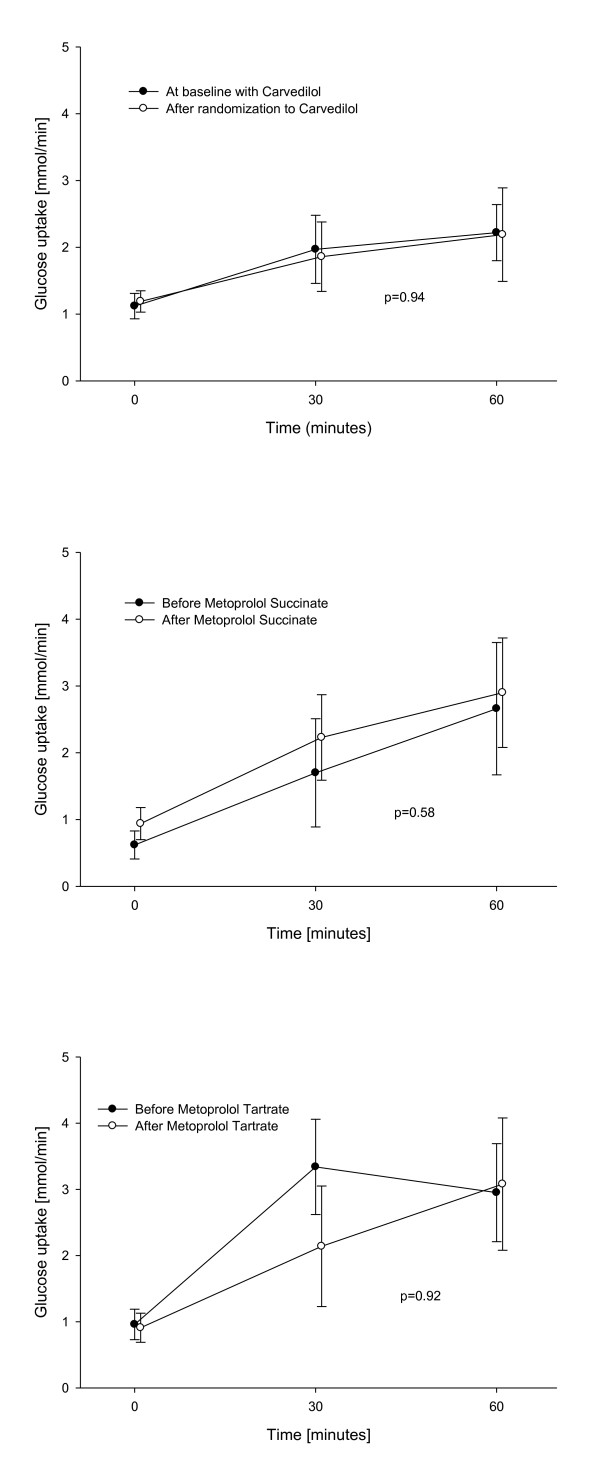
**Forearm glucose uptake**. Forearm glucose uptake during insulin infusion, before and after the treatment period with carvedilol, metoprolol succinate and metoprolol tartrate respectively.

The group of patients with HF had impaired forearm glucose uptake compared to healthy individuals (Figure 4). During insulin infusion, forearm glucose uptake in the carvedilol group was similar throughout the study: Glucose uptake after 30 minutes of insulin infusion, increased from 1.12 mmol/min ± 0.19 to 1.97 mmol/min ± 0.51 and to 2.22 mmol/min ± 0.42 after 60 minutes of infusion on the first examination day with a similar increase seen on the second examination day with an forearm glucose uptake of 1.19 mmol/min ± 0.16 to 1.86 mmol/min ± 0.52 after 30 minutes and 2.19 mmol/min ± 0.69 after 60 minutes (p = 0.94 between the two days) (Figure 5a). Switching carvedilol to metoprolol succinate or to metoprolol tartrate did not affect glucose uptake either, as it is shown in Figures 5b and 5c, (p = 0.58 between treatment with carvedilol and metoprolol succinate and p = 0.92 between treatment with carvedilol and metoprolol tartrate).

As expected, serum-insulin concentration increased in the infused arm during insulin infusion and this increase did not change between treatment groups or from day 1 to day 2 (data not shown). Systemic serum-insulin concentrations were kept constant during the intra-arterial insulin infusion and did not change from day 1 and 2 in either of the three treatment groups.

### Ambulatory 24 hour blood pressure and pulse measurement

**Figure 6:**
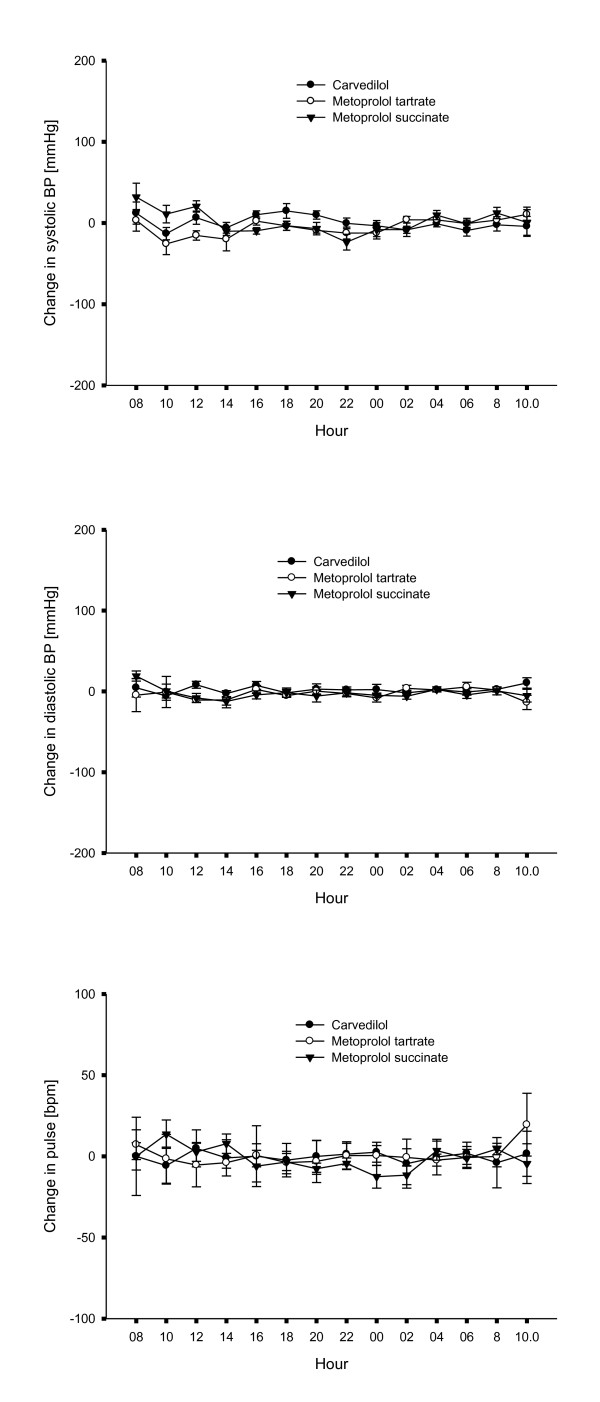
**Blood pressure and pulse**. Differences in systolic blood pressure, diastolic blood pressure and heart rate before and after the two month treatment period with carvedilol, metoprolol succinate and metoprolol tartrate respectively.

Data on systolic and diastolic blood pressure and heart rate on the two examination days during ambulatory 24-hour blood pressure measurement and heart rate measurement are presented in Figure 6. Both systolic, diastolic blood pressure as well as heart rate measurements were comparable in the three treatment groups at the beginning of the study and did not change two months after switching beta blocker treatment from carvedilol to metoprolol succinate or metoprolol tartrate.

## Discussion

In this study we examined vascular and hemodynamic effects of changing beta blocker treatment from carvedilol to metoprolol succinate or metoprolol tartrate in patients with documented heart failure. In our study, endothelial function was not changed from switching beta blocker treatment from carvedilol to either metoprolol succinate or metoprolol tartrate. Previous studies have indicated that treatment with carvedilol could improve endothelial function in patients with coronary artery disease [13] and in patients with dilated cardiomyopathy [14]. Studies have shown that endothelial function is associated with NYHA class [15]. In our study all patients were stable with mild heart failure NYHA I-II, also after changing treatment with beta blockers. This fact that patients had mild HF, could have influenced the fact that we were not able to demonstrate a change in endothelial function after switching the beta blocker treatment from carvedilol to metoprolol, neither the succinate or tartrate formulation (Figure 2). Most patients in this study were treated with both statins as well as ACE inhibitors or AT II antagonists known to improve endothelial function in patients with heart failure [16,17]. The fact that we were not able to demonstrate an impaired endothelial function in patients with HF, could be explained by the concomitant medication, known to improve endothelial function. The concomitant medication could impact endothelial function throughout the study period and influence the fact that we were not able to demonstrate a difference in endothelial function after changing beta blocker treatment from carvedilol to metoprolol. Also the fact that co-existence of diabetes is associated with blunted endothelial function [15] might have influenced the results of our study. In the group of patients randomized to a treatment with either carvedilol or metoprolol succinate there were three patients with co-existing diabetes in each group, whereas there were no patients with diabetes in the group randomized to a treatment with metoprolol tartrate. Even though, we found no change after multivariable analysis including co-existence of diabetes.

HF can be regarded as an insulin resistant state with increased risk of diabetes [18]. We have found that the studied group of patients with HF were vascular insulin resistant and had a reduced forearm glucose uptake when compared to healthy controls Beta blockers, as a group, were initially thought to worsen metabolic glucose control, but there have been shown important differences between individual beta blockers. In The Glycemic Effects in Diabetes Mellitus: Carvedilol-Metoprolol Comparison in Hypertensives study (GEMINI), patients with diabetes mellitus (DM) and hypertension treated with metoprolol tartrate had a deteriorated metabolic control when compared to patients treated with carvedilol [7]. In the COMET study more cases of new-onset diabetes occurred in the metoprolol group compared to the carvedilol group [8]. In this study we found no difference in either insulin-stimulated vasodilatation or forearm glucose uptake when changing patients from carvedilol to neither metoprolol tartrate nor metoprolol succinate treatment (Figure 3). From the result of our study we could not find a vascular reaction as the explanation for the changes observed in the COMET study between treatment with metoprolol and carvedilol. In a previous study we found that patients with type 2 diabetes had impaired insulin stimulated vasodilatation when treated with metoprolol compared to those treated with carvedilol [9]. The HF patients were already treated with carvedilol as we found that they were vascular insulin resistant, but changing this treatment did not change the result. We did not see any systemic changes in metabolic control either.

Different groups of beta blockers might have different effects on blood pressure and heart rate. We therefore did a 24-hour ambulatory blood pressure and heart rate measurement in a subgroup of patients from all three treatment groups. When testing the patients on first carvedilol treatment, since treatment with either metoprolol tartrate or metoprolol succinate, we found no difference between the beta blocker treatment groups. In a study by Sanderson a 24-hour heart rate evaluation was done in patients with HF; they compared treatment with carvedilol and metoprolol tartrate and found no difference in diurnal heart rate [10]. In our study we examined groups treated with either metoprolol tartrate or metoprolol succinate at comparable doses and found no changes in either diurnal blood pressure or heart rate. This indicates that metoprolol tartrate instead of metoprolol succinate did not cause a different effect of beta1-adrenergic blockade at doses chosen in our study. In a direct comparison among patients with HF, no differences in hemodynamic parameters were found either between treatments with metoprolol tartrate or metoprolol succinate at equal doses [19].

### Study Limitation

Our patients were already treated with carvedilol at the first examination of endothelial function. Methodologically, it would have been an advantage to the study if patients were beta blocker naïve at entering the study. We found it unethical to withdraw beta blocker treatment in patients with a proven benefit of treatment and risk of worsening of heart failure symptoms in the situation of withdrawal of beta blocker treatment.

A limitation to the study could be the relative small number of patients included. With 9 patients in each group we were not able to show any difference in endothelial function between different beta blocker treatments. The fact that we did not find any difference in endothelial function could be explained by the short treatment period and a risk of a carry over effect.

## Conclusion

In our study we found no changes in vascular reactivity in patients with mild HF by changing treatment from carvedilol to metoprolol tartrate or metoprolol succinate. Previous studies with metabolic advantages of carvedilol compared to metoprolol were not reflected in our study of forearm glucose uptake; no changes were seen between carvedilol or metoprolol treatment. Diurnal blood pressure and heart rate measurement indicate that treatment with carvedilol and metoprolol, both succinate and tartrate formulation is comparable regarding the beta1-adrenergic blocking effect.

## Competing interests

The authors declare that they have no competing interests.

## Authors' contributions

BF carried out study design, examinations of the patients, data analysis and statistics and drafted the manuscript. TSH, CRM, LK, CTP and HD participated in designing the study, data analysis and statistics and critically revising the manuscript. AMP, BC, JRM and CRM participated in the design of the study and revising the manuscript. All authors have read and approved the final manuscript.
